# Activities critical to success and growth of clinical trials networks. What is needed and how are we doing? An Australian and New Zealand perspective

**DOI:** 10.1186/s13063-023-07709-y

**Published:** 2023-11-04

**Authors:** Megan Sanders, Karen Goulding, Ed Oakley, Donna Reidlinger, Katie M Groom

**Affiliations:** 1 Australian Clinical Trials Alliance, Melbourne, VIC Australia; 2 ANZCA Clinical Trials Network, Monash University, Melbourne, VIC Australia; 3 Department of Emergency Medicine, Royal Children’s Hospital Melbourne, Melbourne, VIC Australia; 4 Departments of Paediatrics and Critical Care, University of Melbourne, Melbourne, VIC Australia; 5 Murdoch Children’s Research Institute, Melbourne, VIC Australia; 6 PREDICT (Paediatric Research in Emergency Departments International Collaborative), Parkville, VIC Australia; 7 Australasian Kidney Trials Network, The University of Queensland, Brisbane, QLD Australia; 8 Maternal and Perinatal Health, Liggins Institute, The University of Auckland, Private Bag 92019, Auckland, 1142 New Zealand; 9 National Women’s Health, Te Whatu Ora, Te Toka Tumai Auckland, Auckland, 1023 New Zealand; 10 PSANZ IMPACT Network – Perinatal Society of Australia and New Zealand Interdisciplinary Maternal Perinatal Australasian Collaborative Trials Network, Mornington, VIC Australia

## Abstract

**Background:**

Clinical trial evidence underpins evidence-based medicine and the improvement of healthcare worldwide. In Australasia, a significant proportion of clinical trials are conducted by geographically dispersed and multidisciplinary clinical researchers under the auspices of Clinical Trials Networks (CTNs). These groups play an important role in contributing to evidence-based medicine, primarily by conducting investigator-initiated clinical trials. Despite their clear benefits in terms of return on investment, CTNs suffer significant challenges.

**Methods:**

We conducted surveys and focus groups with Australian and New Zealand CTNs to identifying the activities and attributes that enable CTNs to operate successfully. Based on our findings, we then conducted further surveys of Australian and New Zealand CTNs to identify the prevalence of these success factors in existing CTNs.

**Results:**

Our focus groups identified three key themes associated with success and growth of a CTN: engaged membership, established infrastructure, and sustainability; and thirteen critical success factors: shared vision and motivation; strong leaders, governance and succession planning; an executive officer; sustainable funding for operations; effective communication; diverse representation and consumer input; transparent processes; a strong pipeline of trials; a reputable and recognised CTN brand; innovation and adaption; an effective group of network sites with a skilled workforce; embedded trials and prioritisation of research. These key themes and the relevant key areas were presented to 30 CTNs. Two factors were almost universally present in CTNs, reflecting the importance of these attributes: the presence of an executive officer, and a strong pipeline of trials. Three factors had a particularly low prevalence: sustainable funding for operations, effective communication, and embedded trials.

**Conclusions:**

By supporting both emerging and established CTNs to achieve critical success factors, we can improve the efficiency of CTNs to continue to contribute and expand their clinical trial activities. Particular focus needs to be on finding sustainable funding for CTNs, and raising awareness of the critical role undertaken by CTNs to improve healthcare and health outcomes.

**Supplementary Information:**

The online version contains supplementary material available at 10.1186/s13063-023-07709-y.

## Background

Evidence-based medicine is critical to improving patient care and health outcomes [1–3]. Underpinning evidence-based medicine is the “individual clinical expertise, the values and desires of the patient, and the best available research” [4]. Such research may be commercially sponsored or publicly funded research. Clinical Trials Networks (CTNs) bring together communities of clinical researchers that are active in defined areas of clinical trials research and often geographically dispersed and multidisciplinary. While some CTNs may facilitate commercially sponsored research, their primary function is to design, conduct, and publish investigator-initiated (and often publicly funded) clinical research. Thus, CTNs play an important role in contributing to evidence-based medicine.

CTNs have a broad and diverse membership encompassing triallists, healthcare providers, and consumer or patient representatives. Through the reach and expertise of their membership, successful CTNs facilitate the delivery of multicentre trials, include peer and consumer review and endorsement processes to develop high-quality patient and clinician relevant clinical trial proposals, have a successful track record of practice-changing trials, have established central and site infrastructure and skilled workforce to support multiple multi-site clinical trials, have capacity for enhanced translation and implementation of clinical trial findings beyond the trial sites, and have a wide variety of stakeholders including international partnerships.

Dependent upon their core activities and responsibilities, CTNs are often defined as either “facilitating” or “coordinating” networks (see Table 1).

**Table 1:** Core activities and responsibilities of Clinical Trials Networks

Activities and responsibilities of “facilitating networks”	Additional activities and responsibilities of “coordinating networks”
Identification of important/priority clinical questions	Direct trial coordination and management
Collaborative study protocol development	Site management
Peer-review and endorsement of trials	Data management
Convene scientific meetings	Central enrolment of trial participants
Grant writing	Trial monitoring
Education/training/mentoring of researchers	Statistical analysis
Advocacy and industry/consumer liaison	Regulatory affairs
	Study sponsor
	Assistance with site selection and trial oversight

The economic benefits of investigator-initiated clinical trials conducted through CTNs have recently been published [5]. In the report, the benefit to cost ratio of investigator-initiated clinical trials was 5.8:1; that is, for every $1 spent on trials in Australia, a return on investment of $5.80 is achieved. Similar economic benefits have been reported internationally, albeit in the context of the cost of trials and not necessarily the cost of the network [6, 7]. This is a remarkable achievement and return on investment for CTNs.

However, the same Australian report noted there remain significant challenges for CTNs, not-the-least their reliance on in-kind support [5]. With this in mind, the Australian Clinical Trials Alliance (ACTA), a national body that supports and represents CTNs, Clinical Quality Registries (CQRs) and Clinical Trial Coordinating Centres (CTCCs) undertook an extensive consultation process with CTNs operating across Australia and New Zealand to identify activities and attributes that enable CTNs to operate successfully (via surveys and focus groups) and then assessed the prevalence of each to support their core remit of maintaining membership to conduct clinical trials to contribute to evidence-based medicine (using surveys). The CTNs were considered as three groups: facilitating, coordinating, and newly established or establishing CTNs.

## Methods

### Aim

The aim of this study was to identify activities critical to success and growth of clinical trials networks and to assess what activities CTNs currently undertake.

### Focus group questions development

An email was sent to the administrative contact of 38 Australian and New Zealand CTNs who were associated with ACTA requesting they review the matrix of activities (see Table 1) and identify any additional structural components or operational activities of CTNs. This matrix was used to develop the focus group question guide. The focus group question guide consisted of semi-structured, open-ended questions, informed by the results of this sector-wide consultation (see Additional file 1: Supplementary Text).

### Focus groups

Three focus groups were convened, one for each of the coordinating, facilitating, and newly established/establishing CTNs. Participation in a focus group was primarily a sample of convenience, with the majority of attendees being based in Melbourne, Australia, although all represented national Australian or bi-national Australian and New Zealand networks. A balance between cancer and non-cancer CTNs was considered, given that cancer CTNs have a dedicated funding source (via Cancer Australia) for some CTN infrastructure. Apart from the newly established/establishing CTNs, all invited CTNs were full members of ACTA. Each CTN Chairperson and Executive Officer was invited to join the focus group.

Focus groups were held in September and November 2018, in Melbourne, Australia. All focus groups were recorded and transcribed verbatim.

### Focus group analysis

Transcripts of focus groups were analysed and relevant points collated under themes by the primary researcher. Themes were refined and discussion points reclassified, where necessary. The results of this thematic analysis were reviewed by a working party to prevent potential bias from occurring due to a sole individual undertaking the thematic coding. Discussions pertaining to clinical trial coordination were not included in the thematic analysis. Leading themes and critical success factors were identified.

### Survey development and implementation for prevalence of success factors in Australasian CTNs

A survey was developed to assess CTNs on the prevalence of thirteen critical success factors identified to operate a CTN successfully. These factors aligned with the findings of our focus groups and international literature on success factors for CTNs. Additional questions were based upon generic organisation capacity surveys covering governance, strategic planning, and succession planning. The survey instrument was approved by the Monash University Human Research Ethics Committee (Project ID 23794), and piloted with two CTNs in February 2020, and a further three CTNs in April 2020. Surveys were sent to all 38 CTNs who were members of ACTA. CTNs were asked to self-assess against a range of questions relevant to each of the 13 success factors (Table 2). Surveys were completed between April and June 2020.

**Table 2:** Thirteen success factors for CTNs

The thirteen identified success factors for CTNs include:
1. shared vision and motivation
2. employment of an executive officer
3. a strong pipeline of trials
4. strong leaders, governance and succession planning
5. diverse representation and consumer input
6. transparent processes
7. a reputable and recognised CTN brand
8. innovation and adaptation
9. an effective group of network sites with skilled site workforce
10. trial prioritisation
11. sustainable funding for operations
12. effective communication
13. embedded trials

### Statistical considerations for survey

No formal sample size calculation was conducted, and participation was limited to CTNs that were members of ACTA. Sub-group analyses compared the self-assessed ratings of “facilitating CTNs” compared to “coordinating CTNs” and “newly established/establishing CTNs” compared to “established CTNs”. All analyses were conducted using Minitab 17.3.1, with values of *p* < 0.05 considered statistically significant.

## Results

### Focus group survey development

Five of 38 CTNs responded with additional items for both structural and operational activities of CTNs. The consolidated list of items appears in Table 3.

**Table 3:** The structural components and operational activities of CTNs identified during sector-wide consultation

	Structural components	Supporting operational activities and resources
Organisational structure	Independently registered company or association sub-entity of a parent organisation Informal entity Registered charity Deductible gift recipient status	Appropriate supporting documentation
	Governance committee	Committee Terms of Reference Meetings
Membership structure	Membership categories	Scientific meeting Education and training workshops Mentoring Communications platforms and policies Membership database
Subcommittee structure	Finance, Audit and Risk Committee	Committee Terms of Reference Meetings Risk management
	Scientific Advisory Committee	Committee Terms of Reference Meetings
	Consumer Advisory Board	Committee Terms of Reference Meetings
Business operations	Finance	Financial software Business Case Report Business continuity plans Funding strategy Budgets
	Human resources	Human resources Operational staff position descriptions
	Legal	Policies Business insurance
	Strategic	Strategic plan Strengths, weaknesses, opportunities and threats analysis
Trial program	Endorsement and prioritisation	Peer review guidelines Authorship policy Endorsement guidelines Research Prioritisation guidelines Presentation guidelines
	Collaborative development of clinical trials	Grant writing guidelines Budget and quoting systems Trial pilot scheme Protocol development
	Consumers	Consumer involvement policy
	Safety oversight	Meetings Committee Terms of Reference Clinical Trials Insurance policy
	Trial management	Standard operating procedures (SOPs)
	Site management	Selection and acquisition procedures Capability assessment
	Research staff: health economics, biostatistics, research translation coordinator, events coordinator, data manager	Business case Position descriptions

### Focus group participants

The “coordinating CTNs” focus group included representatives from five CTNs (Palliative Care Clinical Studies Collaborative, PaCCSC; Australasian Leukaemia and Lymphoma Group, ALLG; Australasian Gastro-Intestinal Trials Group, AGITG; The Australian Kidney Trials Network, AKTN; Australian and New Zealand College of Anaesthetist Clinical Trials Network, ANZCA) with a median of 13 years (range 12 to 47 years) since establishment. There were six attendees (one Chair and five Executive Officers), four of whom attended virtually. The focus group ran for 180 min. The “facilitating CTNs” focus group included representatives from four CTNs (Australasian Stroke Trials Network, ASTN; Australasian Lung Cancer Trials Group, ALTG; Paediatric Research in Emergency Departments International Collaborative, PREDICT; Interdisciplinary Maternal Perinatal Australasian Collaborative Trials Network, IMPACT) with a median of 18 years (14 to 23 years) since establishment. There were five attendees (three Chairs and three Executive Officers), all of those attended in person. This focus group ran for 165 min. The “newly established/establishing CTNs” included three CTNs (Australia and New Zealand Musculoskeletal Clinical Trials Network, ANZMUSC; Australian and New Zealand Alliance for Cardiovascular Trials, ANZACT; Child and Youth Mental Health, CYMH), with three Executive Officers, and was offered virtually. Two CTNs in this focus group had commenced operations during the preceding year, and one had been in operation for 5 years. This focus group ran for 100 min.

### Factors identified as critical to CTN success

**Fig. 1:**
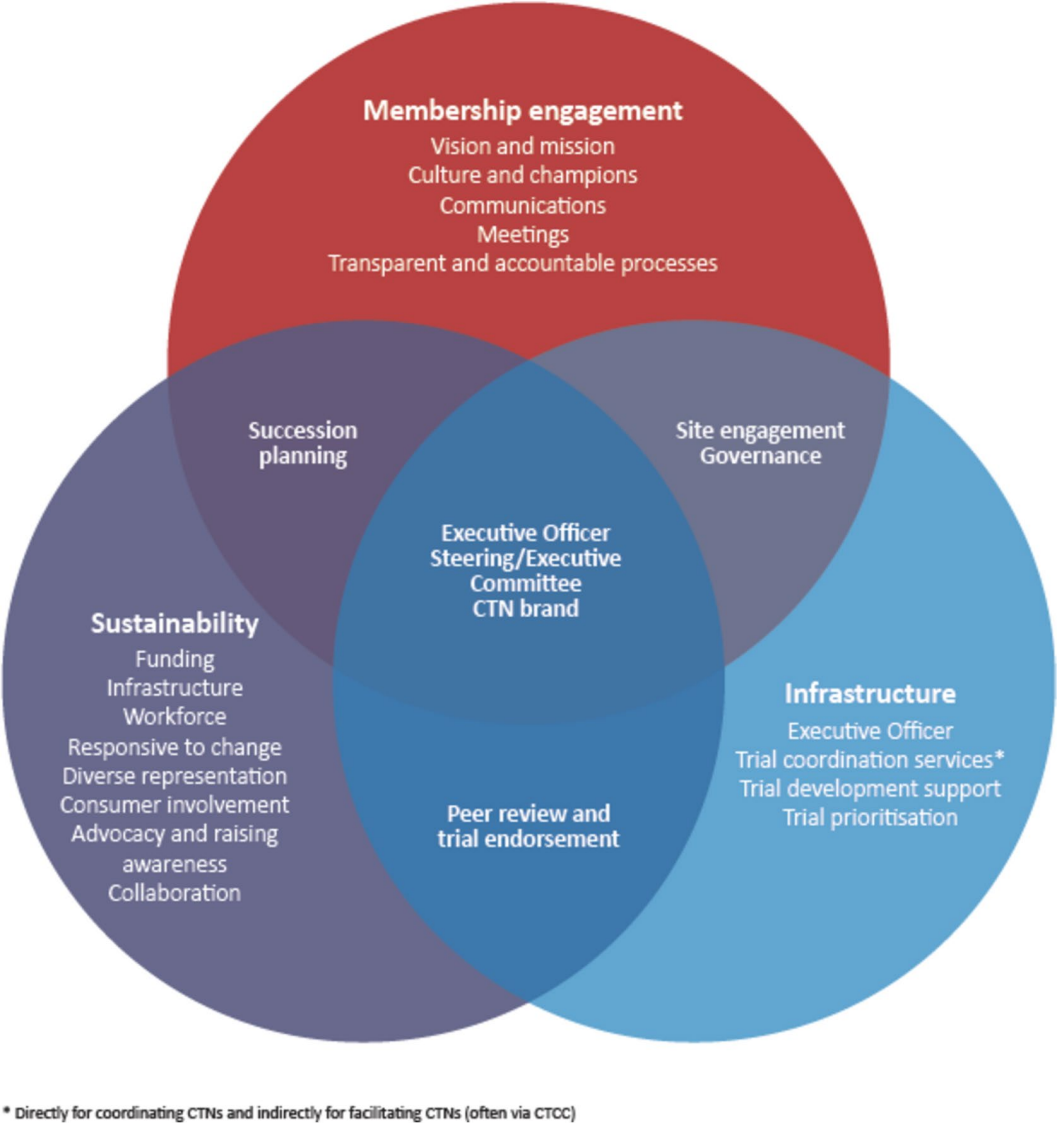
Clinical trials networks themes and activities

Three key themes were identified as critical to CTN success and growth: engaged membership, established infrastructure, and sustainability. Within each theme, key areas were identified (Fig. 1). A number of key tools and resources that supported each theme and area were identified as being critical to CTN operations (Table 4).

**Table 4:** Key tools and resources to facilitate CTN operations

Clinical Trials Network	Description
1. CTN Membership structure	Describes the options CTNs may consider for fees, membership categories, membership approval and meetings of the membership.
2. CTN Governance structure and documents	Describes the options for CTN organisational structures and models, governance frameworks, responsibilities of various committees and considerations for committee Terms of Reference.
3. CTN strategic plan development	Describes the process for developing a CTN mission, vision and strategic plan, and the process for implementing strategic plan objectives and their evaluation.
4. Website	A website shell that can be easily modified by each CTN to include CTN-specific information.
5. Executive Officer duties	Describes the duties of the CTN Executive Officer and provides an editable position description.
6. Trial review, endorsement and prioritisation process	Describes options that a CTN may consider when establishing a clinical trial review and endorsement procedure; to accompany a guidance document on options to determine areas of research prioritisation.
7. Communication strategies	Describes options a CTN may consider in communicating with members and the public.
8. Management of trial metrics—pipeline, active trials, impact of completed trials	A database that keeps a record of clinical trial milestones from development through to publication and impact.
9. Authorship and publication policy	Describes options that a CTN may consider when establishing policy for publication of trials, and the process or criteria for determining authorship.
10. Memorandum of understanding for collaboration with other CTN trials (international and local)	A document covering key considerations and suggested responsibilities when collaborating with another CTN on a clinical trial.
11. Agreement for collaboration with parent organisation	A document covering key considerations and suggested responsibilities if a CTN is a sub- entity of a parent organisation.
12. Standard Operating Procedures (SOPs) for trial management	SOPs for the conduct of multi-site clinical trials (e.g., site activation checklist).
13. Options for funding structures	Description of the opportunities for funding CTN operations and central infrastructure.
14. Roles and responsibilities for network trial Chief Investigator	Allocation of responsibilities and requirements in clinical trials endorsed by the CTN.
15. Safety Committee policy and procedures	Suggested procedures, considerations and template documents for oversight of CTN clinical trial safety.
16. Network meetings and workshops	Description of types of meetings that can be conducted by a CTN.
17. Evaluation of site network capabilities	A process of conducting a needs-analysis for clinical trial sites.
18. Customer relation management database for CTN member management	Database to record CTN member details, and to track communications.
19. Risk management plans for identification and mitigation of risks	Identification of serious risks, development of risk mitigation strategies and procedures for effective management of risks.
20. Formal mentoring structures and processes	Describes the different options a CTN can utilise to undertake mentoring of new Investigators and trialists.
21. Fundraising and marketing plan	Describes fundraising and marketing goals and targets.
22. CTN consumer engagement guidelines	Describes the options CTNs may consider for involvement and engagement of consumers in research-related activities, outlining objectives and commitments for both parties.

#### Engaged membership

The most consistent theme associated with success was an engaged membership:

“One of our enablers is engagement and in broader terms one of our barriers is lack of engagement of investigators”

Member engagement is improved if each member may have the opportunity to become a leader by ensuring governance committee members serve a finite number of terms, and the processes for “election” to governance committees is clear. When engagement is high, key operations flourish, and in a circular relationship, when the CTN is operating successfully, membership engagement remains high. A key factor is the intangible network culture that promotes passion, goodwill, and research.

“I think it’s the culture of the people, the enthusiasm, the goodwill. The network runs 90 percent, like all the other networks, on goodwill of clinicians. Without that it would be non-existent”

Governance is also key, and this typically falls to a small number of members that comprise a Steering or Executive Committee. This group needs to be structured, engaged, and transparent. Tools that facilitate transparent and accountable processes are important. Engagement in CTNs is often driven by champions or Executive Committee members, and their communication with the rest of the membership is critical to maintaining engagement.

Uniting members with a clear vision and mission is also important: typically, this mission is to improve healthcare by generating evidence through clinical trials. The vision and mission of a CTN should be clearly developed with a strategic plan that can be operationalised by governance committees.

“… strong motivation among the members to improve outcomes for their patients. … this is partly because outcomes are so poor for many diseases, that gives that group of physicians a very strong imperative to try and improve outcomes.”

Activities that support member engagement such as scientific meetings, workshops and social events, and communication directly to members were also seen as important. Communication with individual members who are contributing at a high level to the CTN must also be considered.

“It’s crucial to keep that communication going and it’s often at the operational level easy to think things are flowing fairly nicely, but then if investigators are not up to date and they suddenly get a question about X or Y or they get an update that there’s a problem, that can cause issues. Communication is important.”

Social media platforms such as LinkedIn, Twitter, and Facebook, that are not specifically targeted to members, were considered less important for communication as they did not necessarily return high membership engagement. However, when using social media, CTNs felt that different platforms should be targeted for different stakeholder groups, for example, CTN members are most likely to access Twitter, but Facebook is more likely to be effective in raising community awareness. From an advocacy perspective, the CTNs noted that their resources were stretched and suggested “trying to get your message out there among everybody else’s message” remained a barrier in the face of limited resource.

#### An established infrastructure

A CTN requires a structure with effective central network activity that can support and facilitate a pipeline of clinical trials across multiple sites. An Executive Officer is central to operations. The Executive Officer performs a range of duties including supporting governance committees; coordinating events, smaller interest groups and meetings; implementing and measuring strategic objectives; and managing budgets. In addition, they are expected to maintain membership and stakeholder engagement through communication. CTNs must prioritise research, and various methods were employed by CTNs to do this including Delphi processes (a method of formally obtaining consensus), or holding concept development workshops where members indicate support for concepts usually centred around feasibility, lack of existing evidence and unmet need and scientific merit. However, some noted these processes were not perfect.

“We do want to think more strategically about prioritisation though; where are the issues and where are we going to get our greatest value from.”

There were differing models for engaging recruiting sites: either though a site accreditation process or directly with CTN members, who would become clinical trial site principal investigators. CTNs felt there was a burden associated with introducing new sites to a CTN, and their associated processes, which was overcome somewhat in the setting of a coordinating CTN or by a well-resourced central network administration.

“… having the CTN office being able to provide good infrastructure is very helpful because by far the majority of sites don’t have the infrastructure to actually run a multisite trial.”

Trial coordination services were offered by some CTNs, but for others, they relied on the services of the clinical trial coordinating centres.

Consumer engagement was also seen as vital, ensuring the development of patient-relevant clinical trials—however, such engagement is sometimes challenging. Setting up appropriate processes and infrastructure for consumer review is an ongoing need.

“Often the consumers that engage are semi-professional consumers who are representing groups who have very much got their own agenda... I think guidelines or leadership of the consumer group is really important to make them effective.”

#### Sustainability

Most CTNs had a membership that included a variety of diseases and/or disciplines, which enhanced sustainability, as different trials in different areas could be run concurrently, ensuring a pipeline and trial program was maintained, without exhausting the patient population available for clinical trials. Some CTNs had successfully formed partnerships with Clinical Trials Networks overseas, including in Canada, Europe, UK, and Asia.

As the CTNs rely heavily on committed investigators and members for, among other items, clinical trial ideas and conduct, membership engagement and executive oversight, succession planning for key roles is also important and can be achieved through formal mentorship programs and other activities. This is particularly important for early-career researchers who can benefit from the reputation that the CTN has built based on more experienced researchers. This fostering of early-career researchers is also critical to sustainability of the CTN because it ensures a constant flow of new members who can replace the key roles. Reputation of the CTN within the sector can be built by timely completion and publication of high-quality trials, advocacy and awareness, and through collaboration with other networks. Using the CTN name and logo on publications and presentations is an important branding tool that helps build the reputation of the CTN. Peer review to ensure research conducted was of high-quality and maintain the CTN reputation was seen as an important part of the sustainability, including a commitment to publish trial results at the end of the study. Some CTNs reported difficulties with the publication process, and control of timely publication by members remains an ongoing issue for many CTNs. Various approaches to writing committees were being taken, and the CTNs noted that establishing an authorship policy upfront is important.

A strong and effective pipeline of trials ensures an ongoing portfolio of studies that allows infrastructure to be maintained and retention of key experienced staff. Some CTNs had established processes for piloting clinical studies, and being able to support such studies to demonstrate feasibility was seen as an effective way to enhance success in a major project.

Lack of resources, especially financial ones, remains a significant barrier, with several CTNs noting the lack of appropriate remuneration for trial conduct impacts the workload of site trial coordinators, which in turn hampers engagement with CTNs. A potential solution would be to share trial coordinators between clinical areas at sites, which would ensure better resource utilisation, and justify long-term employment for the coordinator. However, there are limitations to this including study funding, prioritisation, requirements for specific knowledge/experience, and employment arrangements. Others felt that embedding trials into the healthcare system may provide a solution to this resourcing problem, and providing a career pathway for research staff would aid in retention.

“Trials shouldn’t be an add-on to good clinical environments and yet they still are; from the staffing that are at the trial sites, the way the hospitals engage in terms of governance processes, etcetera. It’s still very much an add-on. I’d like to see it become more of core business … with some cost recovery on the hospital side from their participation in trials as part of their core business.”

Funding remains a major issue for sustainability, with few CTNs reporting certain long-term funding. Some have been involved in fundraising efforts, but the investment for this was significant. Alignment with relevant professional societies and medical colleges provided some CTNs with financial support, but this was felt to be more challenging for CTNs with a multidisciplinary membership.

Lastly, the CTNs noted they must remain agile and adaptable, given the evolving clinical trial environment.

### CTG prevalence survey respondents

Of the 38 CTNs invited to participate, 30 CTNs completed the survey (response rate 79%). The majority of CTNs were established prior to 2017 (20/30, 66%). “Facilitating CTNs” represented 60% (18/30) with “coordinating CTNs” 40% (12/30).

### CTG prevalence survey results

Results of the prevalence survey are reported in Table 5. Typically, the CTNs had memberships that included a variety of disciplines, and most (87%) provided opportunities for consumer involvement and engagement. Emerging CTNs were less likely to describe their strategic plan as good or adequate compared to established CTNs (50% vs 86%), and coordinating CTNs were more likely to have a strategic plan compared to facilitating CTNs (100% vs 56%), and describe their plan as good or adequate (92% vs 50%). Succession planning was less established in emerging CTNs compared to established CTNs (40% vs 80%). While most CTNs employed an Executive Officer, the funding sources used were variable—the predominant source of funding was grant funding (27%), or grant funding supported by other sources (13%). Only 7% reported funding was via membership fees. Operational funding was via research grants (47%), government sources (40%), philanthropic funding (27%), or parent organisations (23%). Only 10% of CTNs received funding from industry or commercial sponsors. Almost half of respondents (47%) reported that they had no ongoing operational funding.

**Table 5:** CTN prevalence survey results

	All (*n* = 30)	Emerging (*n* = 10)	Established (*n* = 20)	Coordinating (*n* = 12)	Facilitating (*n* = 18)
**1. Shared vision and motivation**	**81%**	**74%**	**85%**	**95%**	**71%**
Has a vision and/or mission statement	27 (90%)	8 (80%)	19 (95%)	12 (100%)	15 (83%)
*Mission statement is written and clear and specific/reasonably specific*	*23 (85%)*	*5 (63%)*	*18* (*95%)*	*11* (*92%)*	*12* (*80%)*
Has a strategic plan	22 (73%)	8 (80%)	14 (70%)	12 (100%)	10 (56%)^b^
*Strategic plan is written well/adequately and is regularly updated/may require updating*	*16 (73%)*	*4 (50%)*	*12* (*86%)*	*11* (*92%)*	*5* (*50%)*
Regular governance meetings at least twice per year, with quorum; reasonably effective and documented	28 (93%)	9 (90%)	19 (95%)	12 (100%)	16 (89%)
Governance committees are documented, adequate, are usually followed and have reasonably clear roles	29 (97%)	10 (100%)	19 (95%)	12 (100%)	17 (94%)
Has a risk management plan	12 (40%)	3 (30%)	9 (45%)	9 (75%)	3 (17%)^b^
*Risk management plan is written and clear/reasonably clear and specific/reasonably specific*	*12 (100%)*	*3 (100%)*	*9* (*100%)*	*12 (100%)*	*3* (*100%)*
**2. Strong leaders, governance and succession planning**	**20 (67%)**	**4 (40%)**	16 (80%)*	**10 (83%)**	**10 (56%)**
**3. An Executive Officer**	**86%**	**78%**	**90%**	**91%**	**82%**
Has an Executive Officer	24 (80%)	8 (80%)	16 (80%)	11 (92%)	13 (72%)
*Executive Officers’ duties clearly described*	*22 (92%)*	*6 (75%)*	*16* (*100%)*	*10 (91%)*	*12* (*92%)*
**4. Sustainable funding for operations**	**15%**	**6%**	**21%**	**26%**	**12%**
More than 60% of operational funding provided on an ongoing basis	5 (21%) *n* = 24	1 (13%) *n* = 8	4 (25%) *n* = 16	2 (18%) *n* = 11	3 (23%) *n* = 13
Has a fundraising plan	1 (10%) *n* = 10	0 (0%) *n* = 4	6 (17%) *n* = 6	1 (33%) *n* = 3	0 (0%) *N* = 7
**5. Effective communication**	**45%**	**33%**	**51%**	**58%**	**36%**
Clearly defined membership	22 (73%)	6 (60%)	16 (80%)	9 (75%)	13 (72%)
Effective customer relationship management system to record membership information	14 (47%)	3 (30%)	11 (55%)	6 (50%)	8 (44%)
Tools to help member satisfaction and engagement	8 (27%)	2 (20%)	6 (30%)	6 (50%)	2 (11%)^b^
Written communication strategy	10 (33%)	2 (20%)	8 (40%)	7 (58%)	3 (17%)^b^
**6. Diverse representation and consumer input**	**56%**	**54%**	**58%**	**73%**	**46%**
Membership includes a variety of disciplines and diseases relevant to the scope of the CTN	24 (80%)	9 (90%)	15 (75%)	8 (67%)	16 (89%)
Consumer representation on governance or other network committees	19 (63%)	7 (70%)	12 (60%)	10 (83%)	9 (50%)
Consumer participation in a consumer advisory panel	14 (47%)	2 (20%)	12 (60%)	9 (75%)	5 (28%)
Consumer input into clinical trial consent procedures	14 (47%)	5 (50%)	9 (45%)	8 (67%)	6 (33%)
Consumer involvement in prioritisation of research	14 (47%)	4 (40%)	10 (50%)	9 (75%)	5 (28%)
**7. Transparent process**	**55%**	**50%**	**57%**	**72%**	**43%**
Terms of reference for all CTN committees	26 (87%)	10 (100%)	16 (80%)	10 (83%)	16 (89%)
Pre-defined priority areas for clinical trial development	12 (40%)	3 (30%)	9 (45%)	9 (75%)	3 (17%)^b^
Pre-defined priority areas for allocation of CTN resources	13 (43%)	4 (40%)	9 (45%)	9 (75%)	4 (22%)^b^
Authorship guidelines	14 (47%)	4 (40%)	10 (50%)	7 (58%)	7 (39%)
Endorsement criteria	17 (57%)	4 (40%)	13 (65%)	8 (67%)	9 (50%)
**8. A strong trial pipeline of trials**	**26 (87%)**	**6 (60%)**	**20 (100%)**^a^	**12 (100%)**	**14 (78%)**
**9. A reputable and recognised CTN brand**	**72%**	**71%**	**73%**	**90%**	**61%**
Maintains a database/spreadsheet to track clinical trial milestones	18 (60%)	6 (60%)	12 (60%)	11 (92%)	7 (41%)^b^
Has research endorsement guidelines	18 (60%)	5 (50%)	13 (65%)	10 (83%)	8 (44%)
Updates website quarterly	25 (86%) *n* = 29	8 (89%) *n* = 9	17 (85%)	11 (92%)	14 (82%)
Updates social media quarterly	20 (83%) *n* = 24	6 (86%) *n* = 7	14 (82%) *n* = 17	10 (92%)	9 (75%)
**10. Innovation and adaption**	**21 (70%)**	**5 (50%)**	**16 (80%)**	**11 (92%)**	**10 (56%)**^**b**^
**11. An effective group of network sites with skilled site workforce**	**53%**	**56%**	**50%**	**NR**	**53%**
Evaluations of network site capability and capacity	5 (28%) *n* = 18	2 (22%) *n* = 9	3 (33%) *n* = 9	NR	NR
Interacts with sites at least quarterly	14 (78%) *n* = 18	8 (89%) *n* = 9	6 (67%) *n* = 9	NR	NR
**12. Embedding trials**	**37%**	**40%**	**35%**	**52%**	**26%**
Has capacity to undertake multi-site trials	20 (67%)	6 (60%)	14 (70%)	10 (83%)	10 (56%)
Run trials that use standard of care visit schedules for data collection points	13 (43%)	5 (50%)	8 (40%)	8 (67%)	5 (28%)
Has partnerships with hospitals with memorandums of understanding relevant to embedding trials	7 (23%)	3 (30%)	4 (20%)	4 (33%)	3 (17%)
Have a documented strategy for trial embedding	4 (13%)	2 (20%)	2 (10%)	3 (25%)	1 (6%)
**13. Prioritisation of research**	**21 (70%)**	**6 (60%)**	**15 (75%)**	**10 (83%)**	**11** (**61%)**

Where multiple questions were asked, the bolded percentage represents the average percentage of sub-questions. Indented questions use the denominator of positive responses in the preceding question

*NR* not reported

^a^Denotes statistically significant difference between emerging and established CTNs

^b^Denotes statistically significant difference between coordinating and facilitating CTNs

One in three CTNs reported having a written communication strategy, but there were differences between emerging vs established and coordinating vs facilitating CTN subgroups. In terms of establishing a reputable and recognised brand, the most commonly reported measures were a website and social media updates. Reputation was also supported by regular monitoring of CTN milestones. Importantly, there was no difference observed between established and emerging CTNs, although coordinating CTNs were much more likely to undertake monitoring of clinical milestones compared to facilitating CTNs (92% vs 39%).

Collaborations with other CTNs either internationally, or on a cross-disciplinary basis to contribute to the pipeline of clinical trials was common (90%), but was more common in established (100%) than emerging CTNs (60%) and in coordinating than facilitating CTNs (100% vs 75%). Emerging CTNs were also more likely to interact with sites regularly compared to established CTNs (89% vs 67%).

Most CTNs reported good or adequate structures for responding to change, with better adaptive capacity observed in more established or coordinating CTNs. Embedding trials into standard practice has the ability to simplify data collection, and this was reported by 13 CTNs, seven CTNs reported having partnerships with hospitals to embed trials, but only four CTNs had a documented strategy for embedding trials.

Trials are prioritised by informal discussions within the CTN (80%) or through concept development workshops (57%). Just under one-half of CTNs used an annual strategic prioritisation process (40%), while just under one-quarter (23%) reported using a Delphi process.

## Discussion

Our study has identified themes of success for CTNs, the prevalence of these success factors, and the tools which could operationally support them. Following this research, ACTA has developed a toolkit that aims to support the work of CTNs (see https://clinicaltrialsalliance.org.au/resources/). Identifying and developing tools to support the critical operations for success is a key foundation for maximising efficiency and effectiveness in the sector.

In this study, we engaged with the CTNs to uncover the factors they thought were necessary for success. It remains to be seen whether these factors are those that are actually useful. Of the 13 key factors critical for success of CTNs, we found three that had particularly low prevalence: sustainable funding for operations, effective communication, and embedding trials. Sustainable funding remains problematic for CTNs, with most CTNs relying on research grants for the majority of their operations.

Clinical research underpins evidence-based medicine, which in turn leads to better healthcare. However, clinical trials may fail for a number of reasons outside of safety and efficacy [8]. Just over one-fifth of phase 3 studies fail due to lack of funding, and a significant number of trials are underfunded [8]. This lack of funding impacts not only on the ability to complete studies but also on ethics given patients may be recruited into a study that never completes. A key component of sustainable clinical trial funding is the inclusion of a budget to sufficiently resource for clinical trial development and multi-site infrastructure, such as CTNs. Alternative funding streams, such as direct hospital or government funding, are urgently needed to ensure the sustainability of the CTN sector in Australia. With a return shown to be 5.8:1, this funding would be a wise investment [5]. In the absence of this, CTNs are left to resort to fundraising or collaboration with international clinical trials or commercial clinical trials.

Whether an engaged membership is a result of or a cause of a successful CTN remains difficult to distinguish and perhaps may be a circular relationship. Despite this, engagement must be a focus at the outset as membership engagement is critical to the success of CTNs, with communication vital to ensuring their ongoing engagement. So, while effective communication is important for success, the effectiveness of both internal and external communication was reportedly poor. This may be due to the reliance on voluntary goodwill to produce the CTN communications often through newsletters and social media. Communication within a trial is part of the coordination of the trial itself. For coordinating CTNs, others have reported that communication in any trial setting is critical and is particularly so in studies that are spread across different states [9]. Support of clinical trial sites can be maintained through centralised training and weekly “frequently asked questions” sessions [9]. Relationship building between investigational sites and the network can improve the quality of work [10]. Clear and concise communication aids this relationship [10]. It is important that any messaging from the CTN to its trial sites or indeed any of its stakeholders is consistent, transparent, and organised [10].

Embedding trials into clinical practice also had poor prevalence, although this may be explained, at least in part, by a lack of understanding on the respondent’s part about how this is done operationally or because there was no documented procedure for how this was done. It was unclear whether this is a critical factor or an aspirational goal of CTNs and would certainly be reliant on policy, systems, services, management, and clinician buy-in beyond the CTN environment. However, given the focus on translational research, this would appear to be an appropriate strategic goal. Implementation of clinical trial results into practice at sites involved with the trial would also be more successful if trials are embedded, allowing sites and workforces to already be familiar and set up for practice change.

Notably, there were differences in the prevalence of key success factors between emerging and established CTNs. Consistently lacking in emerging CTNs were sustainable funding, an effective group of network sites, embedded trials, and effective communication. Our research has not yet identified how CTNs prioritise their activities; however, it appears there are translational issues that influence an individual CTN’s performance and, that over time, CTNs consolidate their systems and processes. Further, coordinating CTNs had high prevalence for seven of the 13 factors identified, while facilitating CTNs had high prevalence on only two of 13 factors (the presence of an executive officer and collaboration for a strong pipeline of trials—suggesting these two are possibly the most critical factors associated with success). Accordingly, the different roles of CTNs should be considered when interpreting differences in the prevalence of the critical success factors. Alternatively, it may reflect that facilitating CTNs tended to be younger and still establishing many of the critical factors. Those that had been established may simply be of higher priority.

There are a number of limitations in the prevalence survey. Firstly, the survey was designed to include factors that the involved CTNs thought were the most important factors and not what have been shown to be the most important, and as such, many of the factors may not actually be critical to success in some instances. It was a once-off survey, and in isolation, there are limits on the interpretation that may be drawn from a snapshot assessment. Repeating the survey annually or biennially may help identify more clearly the prevalence of success factors, assess whether those emerging CTNs show increased prevalence of success factors over time, and determine what success factors appear to be important at different stages of evolution of a CTN or for different types of CTNs. A limitation of the focus groups was that while the 13 key success factors were identified using a robust focus group approach, it would have been useful to explore whether the critical success factors differed between coordinating and facilitating CTNs. Given the majority of participants were from Melbourne, Australia, there may be limits to how generalisable the findings are for the CTNs across Australia and New Zealand.

CTNs play an important role in clinical trials research. By supporting both emerging and established CTNs to achieve critical success factors that influence success, we may be able improve the efficiency of CTNs. By continuing or expanding their clinical trial activities, which have already been established to be significant return on investment, we will improve evidence-based medicine and quality healthcare [5]. Particular focus needs to be on finding sustainable funding for CTNs and raising awareness of the critical role undertaken by CTNs to improve healthcare and patient outcomes. The Australian Clinical Trials Alliance is currently raising awareness and providing evidence for the cost-effectiveness of these networks to advanced evidence-based medicine.

### Supplementary Information

**Additional file 1: Supplementary Text.**

## Abbreviations

ACTA: Australian Clinical Trials Alliance
AGITG: Australasian Gastro-Intestinal Trials Group
AKTN: The Australian Kidney Trials Network
ALLG: Australasian Leukaemia and Lymphoma Group
ALTG: Australasian Lung Cancer Trials Group
ANZACT: Australian and New Zealand Alliance for Cardiovascular Trials
ANZCA: Australian and New Zealand College of Anaesthetist Clinical Trials Network
ANZMUSC: Australia and New Zealand Musculoskeletal Clinical Trials Network
ASTN: Australasian Stroke Trials Network
CQR: Clinical quality registries
CTCC: Clinical Trial Coordinating Centres
CTN: Clinical Trials Networks
CYMH: Child and Youth Mental Health
IMPACT: Interdisciplinary Maternal Perinatal Australasian Collaborative Trials Network
PaCCSC: Palliative Care Clinical Studies Collaborative
PREDICT: Paediatric Research in Emergency Departments International Collaborative
SOP: Standard operating procedures
