# Supplementary material for: Activities critical to success and growth of clinical trials networks. What is needed and how are we doing? An Australian and New Zealand perspective
Source: Trials. 2023 Nov 4;24:707. doi: 10.1186/s13063-023-07709-y (PMC10625692; doi:10.1186/s13063-023-07709-y)
Supplement: Supplementary file 1 — Additional file 1: Supplementary Text. [file 13063_2023_7709_MOESM1_ESM.docx]

**SUPPLEMENTARY TEXT**

**Focus group questions for coordinating and facilitating CTNs**

1. Identify one enabler and one barrier to running your CTN as efficiently and as effectively as you would like.
2. Identify and describe essential tools that will allow a network to operate effectively.
3. What makes your network successful?
4. Describe the factors that help shape the goals of your network.
5. If you had to apply blue-sky thinking, how would your network look if there were no limitations to growth?
6. Tell us about the governance structure of your network and what works well.
7. Talk about the ‘culture’ that your network strives to promote.
8. How does your network ensure different stakeholders feel engaged?
9. Can you describe which of these activities are essential to the effective running of your network?
10. How does the network decide which of these activities to undertake?
11. Describe some other operational activities that you undertake that could be made more efficient or effective.
12. Describe any opportunities for your network and others to share resources or services to facilitate effective operations.
13. Describe any key roles or processes that strive towards ensuring your network is sustainable now and for the future

**Focus group questions for establishing and newly established CTNs**

1. Describe the activities/elements/plans that you feel will make your network successful and the work you have undertaken or are planning to undertake to achieve these.
2. What other activities and processes did you undertake to establish your network?
3. Can you comment on ACTA’s facilitation of the CTN establishment? What could have been done better?
4. Describe your plans for sustainability.
5. Describe any challenges and how you overcame them.
6. Talk about the culture that your network strives to promote.
7. How does your network work to ensure different stakeholders feel engaged?
8. Describe any opportunities for your network and others to share resources or services to facilitate effective operations.
